# A cross-sectional study on the long-term impact of COVID-19: Symptoms, disability and daily functioning

**DOI:** 10.4102/hsag.v30i0.2880

**Published:** 2025-07-09

**Authors:** Soraya Maart, Rebecca A. Hofmeyr, Justin J. Muller, Lehlohonlo B. Tserere

**Affiliations:** 1 Department of Health and Rehabilitation Sciences, Faculty of Health Sciences, University of Cape Town, Cape Town, South Africa

**Keywords:** COVID-19, post-COVID syndrome, disability, WHODAS 2.0, long COVID functioning

## Abstract

**Background:**

Evidence suggests that survivors of coronavirus disease 2019 (COVID-19) experience ongoing symptoms, which is known as long COVID, or post-COVID syndrome. Approximately 100 million people globally are experiencing long COVID symptoms. Post-COVID syndrome (PCS) has debilitating effects on functioning and quality of life, potentially qualifying it as a disability.

**Aim:**

This study aimed to assess the impact of PCS on disability levels using the WHO Disability Assessment Schedule (WHODAS 2.0).

**Setting:**

A digital cross-sectional survey was conducted through online platforms, including Facebook, WhatsApp, and Instagram.

**Methods:**

A self-developed questionnaire was distributed via social media to collect demographic information, COVID-19 symptoms, and severity. The WHODAS 2.0 instrument was used to measure disability levels. A total of 101 participants completed the online survey.

**Results:**

Most participants were aged between 21 years and 30 years (66%) and were female (60%). The most common acute COVID-19 symptoms were sore throat, fever, and headaches (84%). Post-COVID symptoms included brain fog and tiredness (82%). There was a statistically significant relationship between brain fog, depression, anxiety, and disability summary scores (*p* < 0.005). The mean WHODAS 2.0 score was 34%, indicating a moderate level of disability.

**Conclusion:**

This study’s results align with previous research, highlighting tiredness and neuropsychiatric symptoms as common among post-COVID patients. Post-COVID syndrome results in moderate disability when assessed using the WHODAS 2.0 with domains of Life Activities and Cognition mostly affected.

**Contribution:**

Post-COVID syndrome should be recognised as a disabling health condition, with rehabilitation prioritised as a critical intervention to enhance functional capacity and quality of life.

## Introduction

Coronavirus disease 2019 (COVID-19) was declared a global pandemic by the World Health Organization (WHO) in March 2020, after it originated in Wuhan, China in December 2019 (Morens et al. 2020). The risk factors associated with contracting the virus with considerable severity were patients with lung diseases, existing heart conditions, diabetes or any immune suppression conditions, obesity and older individuals (Bridwell, Long & Gottlieb 2020). Typical symptoms of COVID-19 are dry cough, malaise, myalgia, headaches, body aches and fever, with some patients reporting loss of smell and taste (Huang et al. 2020). Evidence suggests that COVID-19 survivors could experience continuous symptoms (Han et al. 2022). The longevity of post-COVID symptoms such as persistent exhaustion and a lack of concentration and its ultimate impact on functioning and participation has been reported in other studies (Davis et al. 2021). These prolonged symptoms are known as post-COVID syndrome (PCS) or long COVID. In South Africa, 46.7% of hospitalised and 18.5% of non-hospitalised patients developed long COVID across the Beta, Delta and Omicron waves. It is estimated that there are already approximately 100 million people experiencing long COVID globally, with 0.5 million being South African, making it a public health threat (Perumal, Shunmugam & Naidoo 2023).

Similar to acute COVID-19, the symptoms of PCS are heterogeneous and affects multiple systems (Jennings et al. 2021). Research suggests that the number of pre-existing comorbidities affects COVID-19 symptom resolution (Tenforde et al. 2020). Post-COVID syndrome has been described as symptoms that persist for more than 12 weeks after initial diagnoses (Sudre et al. 2021). Symptoms can include breathlessness, coughing, fatigue, memory and concentration issues, joint pain, dizziness, insomnia, chest pain, depression, and anxiety (Mendelson et al. 2021). According to the WHO (2021), these symptoms can wax and wane, with some people reporting a decrease in one symptom with an increase in another. According to Davis et al. (2021), PCS could easily be recognised as a disability because of the debilitating effects of the symptoms on functioning and quality of life.

The International Classification of Functioning, Disability and Health (ICF) represents disability as interactions between health conditions and contextual factors (WHO 2001). The WHO Disability Assessment Schedule (WHODAS 2.0), a derivative of the ICF has been used by Fettes et al. (2021) to measure functioning and participation in COVID-19 patients. They found that the prolonged physical and social isolation because of the COVID-19 pandemic quarantine increased disability in activities of daily living (ADL) (Fettes et al. 2021). Ninety-six per cent of 201 patients who needed to be isolated for more than 2 weeks because of COVID-19 infection had higher WHODAS average scores, with activities of daily living being the most affected (Fettes et al. 2021). Gaur et al. (2022) similarly reported an increase in physical disability in patients who have recovered from COVID-19 even after 3 months.

A systematic review revealed that fatigue was the most common PCS symptom experienced, with a mean prevalence of 43% and range of 10% – 71% across studies (Jennings et al. 2023). It is then intuitive to assume that many people with PCS will be experiencing difficulties in everyday life. Post-COVID syndrome is not just associated with physical disability, many people have faced discrimination and stigmatisation, thinking that they are still contagious. Evidence confirms that PCS is having a significant impact on the disability burden globally. Thus, understanding the major functioning difficulties of those with PCS might assist with diagnosis and the development of an appropriate care path.

The aim of the study was to describe functioning and participation, among COVID-19 survivors. Specific objectives are as follows:

- To describe the sample in terms of age, highest qualification and sex.
- To describe the COVID-19 and post-COVID symptoms and severity.
- To assess which symptoms contributed to the WHO Disability Assessment Schedule (WHODAS 2.0) disability score.

## Research methods and design

A descriptive exploratory quantitative cross-sectional survey design used as the survey design is convenient for getting large groups of opinions (Allen 2017).

### Sampling

A non-probability sampling approach was used. The questionnaire was shared on social media platforms such as Facebook, WhatsApp and Instagram. Snowballing allowed the questionnaire link to be shared and distributed. Persons who experienced COVID-19 symptoms with or without positive testing were invited to participate. The inclusion without a positive test, would minimise the exclusion of false-negative patients (Wikramaratna et al. 2020). The link was open for 3 months from April to June 2022.

### Sample size calculation

The sample size calculation was based on 57% prevalence of long COVID symptoms (Taquet et al. 2021). A sample of 95 participants was needed to be 95% confident that the results were within a 10% margin of error (Taquet et al. 2021).

### Instrumentation

A self-developed questionnaire was used with the WHODAS 2.0 12-item version. The questionnaire included demographic information, most common symptoms experienced during acute COVID-19, and post-COVID symptoms experienced for more than 12 weeks after the initial onset of COVID-19. Current literature from 2019, 2020 and 2021 informed the development of the symptom lists for acute and long COVID (Davis et al. 2021; Fettes et al. 2021; Huang et al. 2020; Mendelson et al. 2021; Sudre et al. 2021). Face and content validity of the questionnaire were established by asking five staff members who had COVID-19 to complete the questionnaire and to provide feedback on the flow and ease of completing the questionnaire.

### Data management and analysis

Data were exported to Statistica version 13 for analysis. Simple scoring was used to calculate the WHODAS 2.0 summary scores (WHO 2019). A higher WHODAS mean score meant a lower level of functioning and participation. Descriptive statistics were used in the analysis of demographic data, COVID-19 symptoms and PCS symptoms. Pearson’s chi-squared test was used to determine whether there is a significant association between categorical variables with *p* = 0.05. Regression analysis was used to identify post-COVID symptoms contributing to the disability score (dependant variable). Severity of symptoms was scored on a scale from 0 to 5. Summary scores per symptom was calculated and mean values presented.

### Ethical considerations

Ethical approval was obtained from the University of Cape Town Human Research Ethics Committee (HREC) (reference no.:100/2022). The questionnaire was created on Google Forms with an electronic link. All the researchers posted the link on their social media platforms, with the request to share from 01 August 2022 to 08 September 2022. Participants could not proceed with the questionnaire until they had given informed consent. Compliance with the national *Protection of personal information policy* (POPI Act, 2021) was considered, and no individuals were directly targeted during the recruitment. The research study was conducted in accordance with the Declaration of Helsinki (2013).

## Results

A total of 101 participants completed the online questionnaire. Most of the participants were in the age range of 21 years – 30 years (66%) and were female (60%). Seventy-two per cent had a tertiary qualification.

Forty-seven per cent of the sample had contracted COVID-19 more than once. Only two people in the age category 51 years – 60 years required hospitalisation and only one required oxygen therapy during admission (Table 1).

**TABLE 1:** Demographics of the sample (*N* = 101).

Demographics	Frequency	%
**Sex**		
Female	60	59.4
Male	40	39.6
Not disclosed	1	1.0
**Qualification**		
High school	29	28.7
Tertiary	72	71.3
**Age (years)**		
< 20	6	5.9
21–30	67	66.3
31–40	8	7.9
41–50	6	5.9
51–60	12	11.9
61–70	2	1.9
**Number of times contracted COVID-19**		
Once	54	53.5
Twice	37	36.6
> Twice	10	9.9

COVID-19, Coronavirus disease 2019.

### COVID-19 symptoms

**FIGURE 1:**
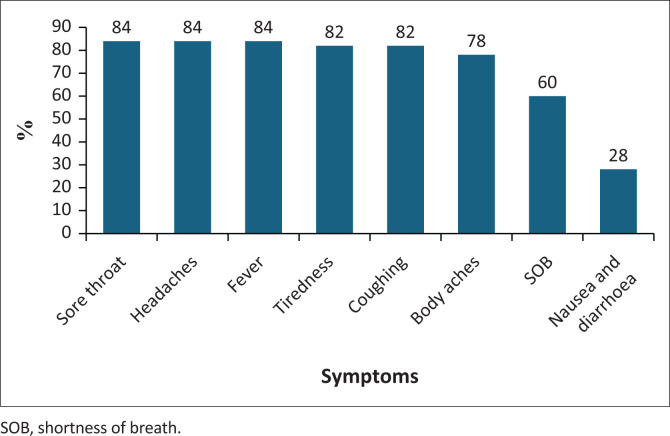
Most common COVID-19 symptoms experienced.

**FIGURE 2:**
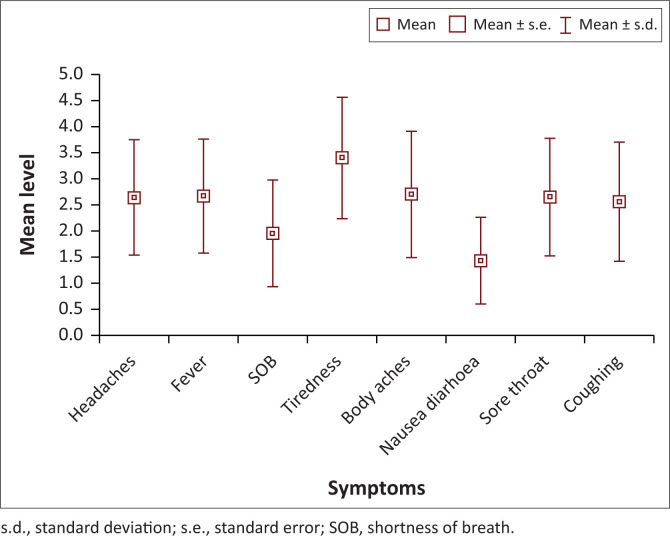
Mean level of severity of COVID-19 symptoms.

The symptoms most experienced were sore throat, fever and headaches (84%), followed closely by tiredness and coughing (82%). Tiredness was experienced as most severe (Figure 1 and Figure 2).

### Post-COVID symptoms

**FIGURE 3:**
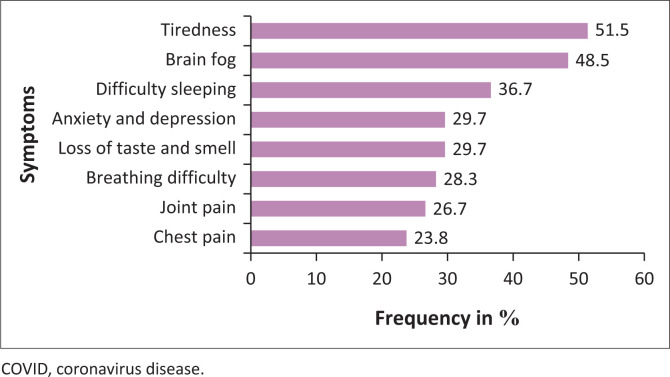
Most common post-COVID symptoms.

Most of the participants had been experiencing post-COVID symptoms for 3 months – 6 months. Noticeably, 12% had been experiencing post-COVID symptoms for > 2 years. Most common and most severe symptoms were tiredness and brain fog (Figure 3).

### Disability assessment – WHODAS 2.0

**FIGURE 4:**
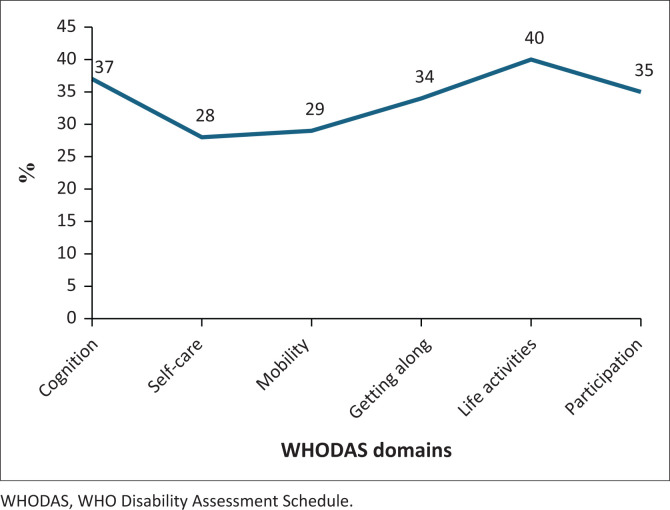
Mean domain scores of the WHODAS 2.0.

The mean WHODAS percentage score was 34% indicating a moderate disability level. The domains of life activities (includes items such as taking care of one’s household, work and school) and cognition that relates to memory, were the most affected domains (Table 2 and Figure 4).

**TABLE 2:** Regression analysis of disability score and post-COVID symptoms (*N* = 101).

Variables	β*	s.e. of β*	*β*	s.e.	*t*(91)	*p*-value
Intercept	-	-	34.030	3.901	8.725	0.000
Tiredness	0.010	0.096	0.148	1.457	0.101	0.919
Brain fog	0.232	0.106	3.337	1.531	2.179	0.032
Difficulty breathing	−0.004	0.112	−0.094	2.578	−0.036	0.971
Joint pain	−0.069	0.094	−1.521	2.072	−0.734	0.465
Loss of taste	0.012	0.088	0.189	1.380	0.137	0.891
Difficulty sleeping	0.037	0.108	0.556	1.627	0.341	0.734
Depression and anxiety	0.511	0.112	7.531	1.654	4.553	0.000
Chest pain	−0.034	0.110	−0.893	2.861	−0.312	0.756

Note: The intercept in a regression model represents the predicted value of the dependent variable (disability score) when all independent variables (post-COVID symptoms) are equal to zero. Adjusted *R*^2^ = 0.35 *F*(8.91) = 7.7. β* denotes the standardised co-efficient whereas only *β* denotes unstandardised co-efficient.

*p* < 0.005.

s.e., standard error; *β*, beta.

Brain fog, depression, and anxiety were predictors of disability. There was a statistically significant relationship between brain fog, depression, anxiety, and the disability summary score, with *p* < 0.005.

## Discussion

This study set out to explore the symptoms of COVID-19 experienced, post-COVID symptoms, its severity and impact on functioning and participation.

The biases introduced by social media recruitment may have influenced the sample, showing a younger population group. However, the results are consistent with previous research which showed high levels of COVID-19 infection and low hospitalisation rates among younger age groups and higher rates of hospitalisation among older adults (Sharma et al. 2020). The demographic distribution of the participants with the majority in the age category of 21 years – 30 years and predominantly female, aligns with trends observed in other studies, where women are more likely to report long COVID symptoms (Duggal et al. 2022) (Table 1). Rudroff, Workman and Bryant (2022) found that women were at greater risk of developing post-COVID symptoms such as fatigue, depression and anxiety than men.

The most frequently presented COVID-19 symptoms were headache, sore throat, fever, tiredness (fatigue) and body aches (Figure 1). This is consistent with other international studies showing fatigue as the most debilitating COVID symptom (Raveendran et al. 2021). Tiredness presented as the most severe COVID 19 symptom among participants, which could likely have persisted as a long COVID symptom.

The PCS is multisystemic with the possibility of recurring and progressive symptoms that should be receiving symptomatic treatment from a multi-professional team (Finterer & Scorza 2022). Researchers have found that among those presenting with long COVID symptoms, fatigue and neuropsychiatric symptoms persisted for longer periods of time (Kim et al. 2022). The findings are consistent with other research that found younger females (< 50) to report greater disability because of fatigue (Sigfrid et al. 2021).

The post-COVID symptoms of brain fog and tiredness were predictors of the WHODAS score, showing a moderate level of disability (Table 2). The domains mostly affected were Cognition and Life Activities. The impact of tiredness and brain fog could have major consequence on the working or studying of young adults. Gaur et al. (2022) using the WHODAS 2.0, showed fatigue and dyspnoea as the most common symptoms persisting 3 months post COVID-19 infection, with significant disability for women in the domain of Getting Around. In a study by Duggal et al. (2022), women were found to be more likely to present with limitations in daily activities post-COVID (Duggal et al. 2022).

## Conclusion

This study found that PCS had a substantial impact on the functioning of participants with the most common symptoms being tiredness and brain fog, which impact the ability to work and perform household tasks. Initial COVID-19 symptom severity could predispose to long COVID disability. Tiredness and brain fog were predictors of moderate disability. Advocacy will be required to recognise PCS as a health condition among a younger population group, requiring appropriate rehabilitation strategies to optimise functioning.

### Implications of research

The research has shown that there is an experience of disability post-COVID-19 infection among young adults. The impact of tiredness and brain fog could impact student academic and work performance. Future research should explore potential treatment strategies, rehabilitation programmes, and the long-term trajectory of recovery to better support those affected by long COVID symptoms.

## References

[CIT0001] Allen, M., 2017, *The Sage encyclopedia of communication research methods*, vols. 1–4, SAGE Publications, Inc., Thousand Oaks, CA.

[CIT0002] Bridwell, R., Long, B. & Gottlieb, M., 2020, ‘Neurologic complications of COVID-19’, *American Journal of Emergency Medicine* 38(7), 1549.e3–1549.e7. 10.1016/j.ajem.2020.05.024 PMC7229718 32425321

[CIT0003] Davis, H.E., Assaf, G.S., McCorkell, L., Wei, H., Low, R.J., Re’em, Y. et al., 2021, ‘Characterizing long COVID in an international cohort: 7 Months of symptoms and their impact’, *EClinicalMedicine* 38, 101019. 10.1016/j.eclinm.2021.101019 34308300 PMC8280690

[CIT0004] Duggal, P., Penson, T., Manley, H.N., Vergara, C., Munday, R.M., Duchen, D. et al., 2022, ‘Post-sequelae symptoms and comorbidities after COVID-19’, *Journal of Medical Virology* 94(5), 2060–2066. 10.1002/jmv.27586 35032030 PMC8958980

[CIT0005] Fettes, L., Bayly, J., De Bruin, L.M., Patel, M., Ashford, S., Higginson, I.J. et al., 2021, ‘Relationships between prolonged physical and social isolation during the COVID-19 pandemic, reduced physical activity, and disability in activities of daily living among people with advanced respiratory disease’, *Chronic Respiratory Disease* 18, 147997312110358–14799731211035822. 10.1177/14799731211035822 PMC8370888 34382888

[CIT0006] Finterer, J. & Scorza, F.A., 2022, ‘A retrospective analysis of clinically confirmed long post-COVID vaccination syndrome’, *Journal of Clinical and Translational Research* 8(6), 506–508. 36452006 PMC9706319

[CIT0007] Gaur, R., Asthana, S., Yadav, R., Ghuleliya, R., Kumar, D., Akhtar, M. et al., 2022, ‘Assessment of physical disability after three months in patients recovered from COVID-19: A cross-sectional study’, *Cureus* 14(1), e21618. 10.7759/cureus.21618 35228971 PMC8874231

[CIT0008] Han, Q., Zheng, B., Daines, L. & Sheikh, A., 2022, ‘Long-term sequelae of COVID-19: A systematic review and meta-analysis of one-year follow-up studies on post-COVID symptoms’, *Pathogens* 11(2), 269. 10.3390/pathogens11020269 35215212 PMC8875269

[CIT0009] Huang, C., Wang, Y., Li, X., Ren, L., Zhao, J., Hu, Y. et al., 2020, ‘Clinical features of patients infected with 2019 novel coronavirus in Wuhan, China’, *The Lancet* 395(10223), 497–506. 10.1016/S0140-6736(20)30183-5 PMC7159299 31986264

[CIT0010] Jennings, G., Monaghan, A., Xue, F., Mockler, D. & Romero-Ortuño, R., 2021, ‘A systematic review of persistent symptoms and residual abnormal functioning following acute COVID-19: Ongoing symptomatic phase vs. post-COVID-19 syndrome’, *Journal of Clinical Medicine* 10(24), 5913. 10.3390/jcm10245913 34945213 PMC8708187

[CIT0011] Jennings, S., Corrin, T. & Waddell, L., 2023, ‘A systematic review of the evidence on the associations and safety of COVID-19 vaccination and post COVID-19 condition’, Epidemiology & Infection 151, e145. 10.1017/S0950268823001279 37594232 PMC10540166

[CIT0012] Kim, Y., Kim, S.E., Kim, T., Yun, K.W., Lee, S.H., Lee, E. et al, 2022, ‘Preliminary guidelines for the clinical evaluation and management of long COVID’, *Infection & Chemotherapy* 54(3), 566–597. 10.3947/ic.2022.0141 36196612 PMC9533168

[CIT0013] Mendelson, M., Nel, J., Blumberg, L., Madhi, S., Dryden, M., Stevens, W. et al., 2021, ‘Long-COVID: An evolving problem with an extensive impact’, *South African Medical Journal* 111(1), 10–12. 10.7196/SAMJ.2021.v111i1.15433 33403997

[CIT0014] Morens, D.M., Breman, J.G., Calisher, C.H., Doherty, P.C., Hahn, B.H., Keusch, G.T. et al., 2020, ‘The origin of COVID-19 and why it matters’, *The American Journal of Tropical Medicine and Hygiene* 103(3), 955–959. 10.4269/ajtmh.20-0849 32700664 PMC7470595

[CIT0015] Perumal, R., Shunmugam, L. & Naidoo, K., 2023, ‘Long COVID: An approach to clinical assessment and management in primary care’, *South African Family Practice* 65(1), 1–10. 10.4102/safp.v65i1.5751 PMC10331047 37427773

[CIT0016] Raveendran, A.V., Jayadevan, R. & Sashidharan, S., 2021, ‘Long COVID: An overview’, *Diabetes & Metabolic Syndrome: Clinical Research & Reviews* 15(3), 869–875. 10.1016/j.dsx.2021.04.007 PMC8056514 33892403

[CIT0017] Rudroff, T., Workman, C.D. & Bryant, A.D., 2022, ‘Potential factors that contribute to post-COVID-19 fatigue in women’, *Brain Sciences* 12(5), 556. 10.3390/brainsci12050556 35624943 PMC9139370

[CIT0018] Sharma, A.K., Ahmed, A., Baig, V.N., Dhakar, P., Dalela, G., Kacker, S. et al., 2020, ‘Characteristics and outcomes of hospitalized young adults with mild covid-19’, *The Journal of the Association of Physicians of India* 68(8), 62–65. 10.1101/2020.06.02.20106310 32738843

[CIT0019] Sigfrid, L., Drake, T.M., Pauley, E., Jesudason, E.C., Olliaro, P., Lim, W.S. et al., 2021, ‘Long Covid in adults discharged from UK hospitals after Covid-19: A prospective, multicenter cohort study using the ISARIC WHO clinical characterization protocol’, *The Lancet Regional Health – Europe* 8, 100186. 10.1016/j.lanepe.2021.100186 34386785 PMC8343377

[CIT0020] Sudre, C.H., Murray, B.H., Varsavsky, T., Graham, M., Penfold, R., Bowyer, R. et al., 2021, ‘Attributes and predictors of long-COVID’, *Nature Medicine* 27(4), 626–631. 10.1038/s41591-021-01292-y PMC7611399 33692530

[CIT0021] Taquet, M., Dercon, Q., Luciano, S., Geddes, J.R., Husain, M. & Harrison, P.J., 2021, ‘Incidence, co-occurrence, and evolution of long-COVID features: A 6-month retrospective cohort study of 273,618 survivors of COVID-19’, *PLOS Medicine* 18(9), e1003773. 10.1371/journal.pmed.1003773 34582441 PMC8478214

[CIT0022] Tenforde, M.W., Kim, S.S., Lindsell, C.J., Rose, E.B., Shapiro, N.I., Clark Files, D. et al., 2020, ‘Symptom duration and risk factors for delayed return to usual health among outpatients with COVID-19 in a multistate health care systems network – United States, March–June 2020’, *MMWR. Morbidity and Mortality Weekly Report* 69(30), 993–998. 10.15585/MMWR.MM6930E1 32730238 PMC7392393

[CIT0023] Wikramaratna, P.S., Paton, R.S., Ghafari, M. & Lourenço, J., 2020, ‘Estimating the false-negative test probability of SARS-CoV-2 by RT-PCR’, *Euro Surveillance* 25(50), 2000568. 10.2807/1560-7917.ES.2020.25.50.2000568 33334398 PMC7812420

[CIT0024] World Health Organization, 2001, *International classification of functioning, disability and health*, World Health Organization, Geneva.

[CIT0025] World Health Organization, 2019, *WHO disability assessment schedule 2.0 (WHODAS 2.0)*, viewed 05 October 2021, from https://www.who.int/classifications/icf/more_whodas/en/.

[CIT0026] World Health Organization, 2021, *A clinical case definition of post COVID-19 condition by a Delphi consensus*, World Health Organization, viewed 05 October 2021, from https://www.who.int/publications/i/item/WHO-2019-nCoV-Post_COVID-19_condition-Clinical_case_definition-2021.1.

